# FieldMaizeLeaf-8: A real-field early vegetative maize leaf stress image dataset from Bangladesh

**DOI:** 10.1016/j.dib.2026.113337

**Published:** 2026-10-09

**Authors:** Laboni Akter, Md Shohel Arman, Sakibul Hasan Chowdhury, Sayed Mohammad Mohsin

**Affiliations:** a Data Science Lab, Department of Software Engineering, Daffodil International University, Daffodil Smart City, Savar, Dhaka, 1216, Bangladesh; b Department of Plant Pathology, Faculty of Agriculture, Sher-e-Bangla Agricultural University, Sher-e-Bangla Nagar, Dhaka, 1207, Bangladesh

**Keywords:** Maize, Zea mays, Leaf disease, Plant pathology, Computer vision, Deep learning, Precision agriculture, Image dataset

## Abstract

Maize (Zea mays L.) is a rapidly expanding cereal crop in Bangladesh, but its yield is threatened by biotic diseases, insect pests, and nutrient-deficiency disorders that are difficult to distinguish visually, particularly at the early vegetative stage. Most publicly available leaf-image datasets used to train computer-vision models for crop diagnosis are captured under controlled laboratory conditions with plain backgrounds, which limits the field-readiness of models trained on them. FieldMaizeLeaf-8 addresses this gap by providing 3975 RGB photographs of maize leaves aged approximately 1–2 months, captured directly in commercial maize fields in Daudkandi, Cumilla, Bangladesh, using a consumer smartphone under natural daylight, background, and viewing-angle conditions, with no laboratory staging. Images are organised into eight classes: one healthy-leaf class and seven stress classes, comprising three biotic conditions (Fall Armyworm damage, Leaf Spot, and Northern Corn Leaf Blight) and four abiotic nutrient-deficiency conditions (Nitrogen, Phosphorus, Potassium, and Magnesium deficiency). Class labels assigned in the field were subsequently reviewed and validated by a plant-pathology expert. The dataset retains natural variability in illumination, leaf orientation, and symptom severity, and is intended to serve as a real-field benchmark for developing and evaluating deep-learning models for maize stress recognition, transfer learning, and explainable artificial intelligence (XAI) applications in precision agriculture.

Specifications Table

**Table:** 

Subject	Computer Sciences
Specific subject area	Field-image classification of biotic and abiotic maize (Zea mays L.) leaf stresses at the early vegetative stage; Image dataset; Computer vision; Deep learning; Artificial intelligence; Precision architecture
Type of data	Image (raw)
Data collection	Images were acquired in situ in smallholder and commercial maize fields, with no laboratory staging, using a Xiaomi 11 Lite NE 5 G smartphone rear camera under ambient outdoor daylight (camera specifications in the Data Description section). Photographs were taken handheld at distances and angles typical of manual crop scouting, preserving natural background, illumination, leaf orientation and symptom severity. Collection took place between 25 January and 20 February 2026, targeting maize plants at the early vegetative stage (∼1–2 months old). Each image was assigned in the field to one of eight classes and subsequently reviewed by a plant-pathology expert; images with ambiguous symptoms or poor quality were relabelled or excluded (workflow in the Experimental Design, Materials and Methods section).
Data source location	Region: Daudkandi Upazila, Cumilla District, Chittagong Division, Bangladesh.
Data accessibility	Repository name: Zenodo Data identification number: DOI 10.5281/zenodo.22162541 Direct URL to data: https://zenodo.org/records/22162541 Instructions for accessing the data: Openly and freely downloadable as a single compressed archive (FieldMaizeLeaf-8.zip, 26.4 GB), no registration required, under a Creative Commons Attribution 4.0 International (CC BY 4.0) licence.
Related research article	None

## 1 Value of the Data

• A real-field, smartphone-acquired alternative to laboratory-style leaf datasets, so models trained or benchmarked on it are more likely to transfer to real deployment such as farmer-facing apps, drone scouting, or advisory kiosks [1,2].
• Combines biotic and abiotic classes within one consistently collected benchmark, suited to fine-grained, explainable classification methods that must distinguish visually similar symptoms requiring different remedies (pesticide/fungicide versus fertiliser correction).
• Enables researchers to train, validate, and benchmark CNNs, vision transformers, and other deep-learning architectures for multi-class stress classification, and to study transfer learning, domain adaptation, and explainable AI (XAI) methods.
• Supports agricultural extension services and precision-agriculture developers in prototyping mobile or edge-deployed maize-stress advisory tools for smallholder farming in Bangladesh and similar regions.
• Provides expert-reviewed, comparatively reliable ground-truth labels for supervised learning and label-noise studies, and can be combined with other public maize datasets [[1], [2], [3], [4]] to study cross-dataset generalisation.

## 2 Background

Maize (Zea mays L.) is now Bangladesh's second most important cereal crop after rice, cultivation expanding rapidly as demand grows from the poultry, dairy, and aquaculture feed industries; the country still imports much of its requirement and seeks higher domestic yields. Realising this depends on timely diagnosis of the biotic and abiotic stresses that most affect maize at the early vegetative stage, when the crop is most vulnerable and corrective action is cost-effective. Fall Armyworm (Spodoptera frugiperda), first detected in Bangladesh in 2018, has spread through the maize belt and can cause 15–73% yield loss if unmanaged [5]; fungal diseases such as Northern Corn Leaf Blight and Leaf Spot likewise cause double-digit losses when diagnosis is delayed [6,7]. Nutrient deficiencies (nitrogen, phosphorus, potassium, magnesium) are widespread on South Asia's poor soils and can visually mimic disease symptoms, causing misdiagnosis [8]. Most public datasets, such as PlantVillage [1], were collected in laboratory conditions and generalise poorly to field imagery [1,2]. Field alternatives such as PlantDoc [2] and the Northern Leaf Blight set [4] lack combined biotic-abiotic coverage and are not South Asian. This dataset closes that gap, combining both stress types from a Bangladeshi smallholder system while preserving natural field variability.

## 3 Data Description

### 3.1 Dataset composition and class distribution

FieldMaizeLeaf-8 comprises 3975 in-field RGB photographs of maize leaves [9], at the early vegetative growth stage (approximately 1–2 months after emergence), distributed across eight classes as summarised in Table 1. Each class is stored in its own image folder within the released archive; folder names correspond to the class names listed below. The subsections that follow describe each class individually, including the underlying biological or agronomic cause of the symptom, the visual criteria used for field-level labelling, and two representative sample images per class.

**Table 1:** Class distribution for FieldMaizeLeaf-8 (n = 3975 images).

Class	Images	% of total	Category
Healthy Leaf	496	12.5%	Healthy (control)
Fall Armyworm Damage	849	21.4%	Biotic – insect pest
Leaf Spot	407	10.2%	Biotic – fungal
Northern Corn Leaf Blight	535	13.5%	Biotic – fungal
Nitrogen Deficiency	425	10.7%	Abiotic – nutrient
Phosphorus Deficiency	500	12.6%	Abiotic – nutrient
Potassium Deficiency	210	5.3%	Abiotic – nutrient
Magnesium Deficiency	553	13.9%	Abiotic – nutrient
Total	3975	100%	–

### 3.2 Challenges during data collection

• **Uneven natural lighting:** variation between sunny and cloudy conditions affected image brightness, contrast, shadowing, and the visibility of leaf symptoms.
• **Visually noisy field backgrounds:** adjacent plants, stems, soil, and weeds created complex, cluttered backgrounds around the target leaf.
• **Overlapping leaves:** dense vegetation sometimes caused neighbouring leaves and stems to overlap, making it harder to isolate an individual leaf clearly.
• **Variation in leaf position and symptom severity:** differences in leaf size, shape, orientation, and disease/deficiency severity required continual adjustment of camera framing and distance.
• **Wind-induced movement:** occasional wind moved leaves during capture, complicating focus and framing.

These conditions were not corrected or filtered out prior to release; they are reflected in the final dataset as a deliberate design choice (see Value of the Data and Experimental Design, Materials and Methods).

### 3.3 Healthy leaf

Healthy leaves form the disease-free control class of the dataset, providing a baseline of normal maize foliar appearance against which every stress class can be visually and computationally compared. At the early vegetative stage, a healthy maize leaf is uniformly green along its full length, with a smooth blade surface, evenly spaced parallel venation, and no chlorotic, necrotic, or discoloured patches. Leaf orientation, turgidity, and a glossy cuticle sheen are additional indicators of good plant health that were considered during field labelling. The absence of any biotic or abiotic symptom is the defining criterion for this class, and it serves as the reference class that a classification model must correctly separate from all seven stress categories. Fig. 1 shows two representative healthy leaves photographed in the field. This class comprises 496 images (12.5% of the dataset).

**Fig. 1:**
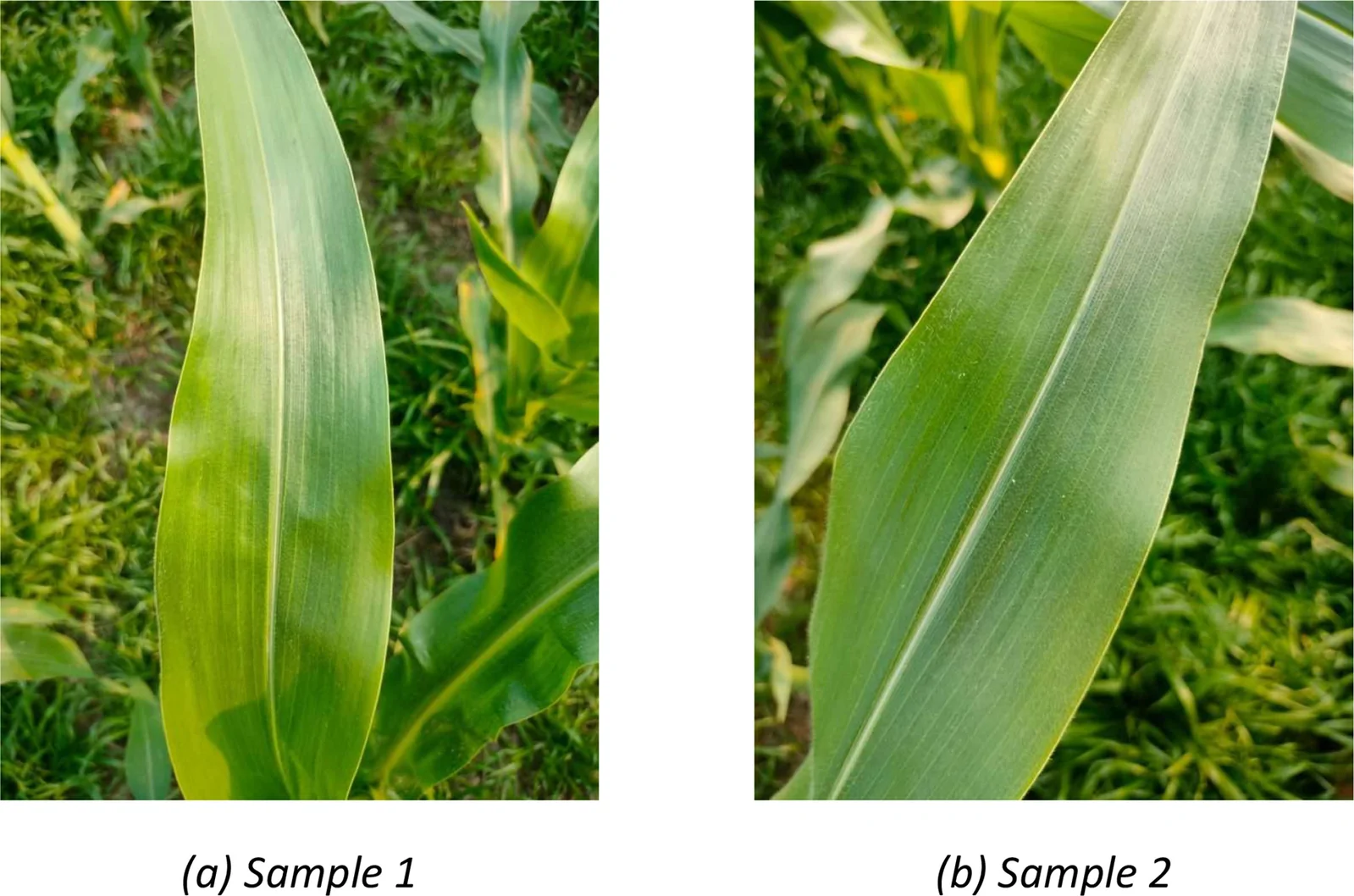
Healthy maize leaves showing uniform green colour and no visible disease or deficiency symptoms.

### 3.4 Fall armyworm damage

Fall Armyworm damage is caused by larvae of the moth Spodoptera frugiperda (J.E. Smith), an invasive lepidopteran pest native to the Americas that was first recorded in Bangladesh in 2018 and has since spread through the country's maize-growing regions, with reported yield losses of 15–73% in untreated fields [5]. Early-instar larvae feed within the unopened whorl, scraping the leaf epidermis to leave translucent “window-pane” patches, while later instars chew irregular, elongated holes through the fully expanded leaf blade and often leave characteristic granular frass near the feeding site. Because larvae feed inside the folded whorl before the leaf unfurls, damage typically appears as a symmetrical row of holes running across the blade once the leaf opens, and is most severe on young leaves near the growing point. Feeding is most active at night, so daytime field inspection typically detects the damage rather than the larvae themselves. Fig. 2 shows two representative examples of Fall Armyworm feeding damage captured in the field. This class comprises 849 images (21.4% of the dataset).

**Fig. 2:**
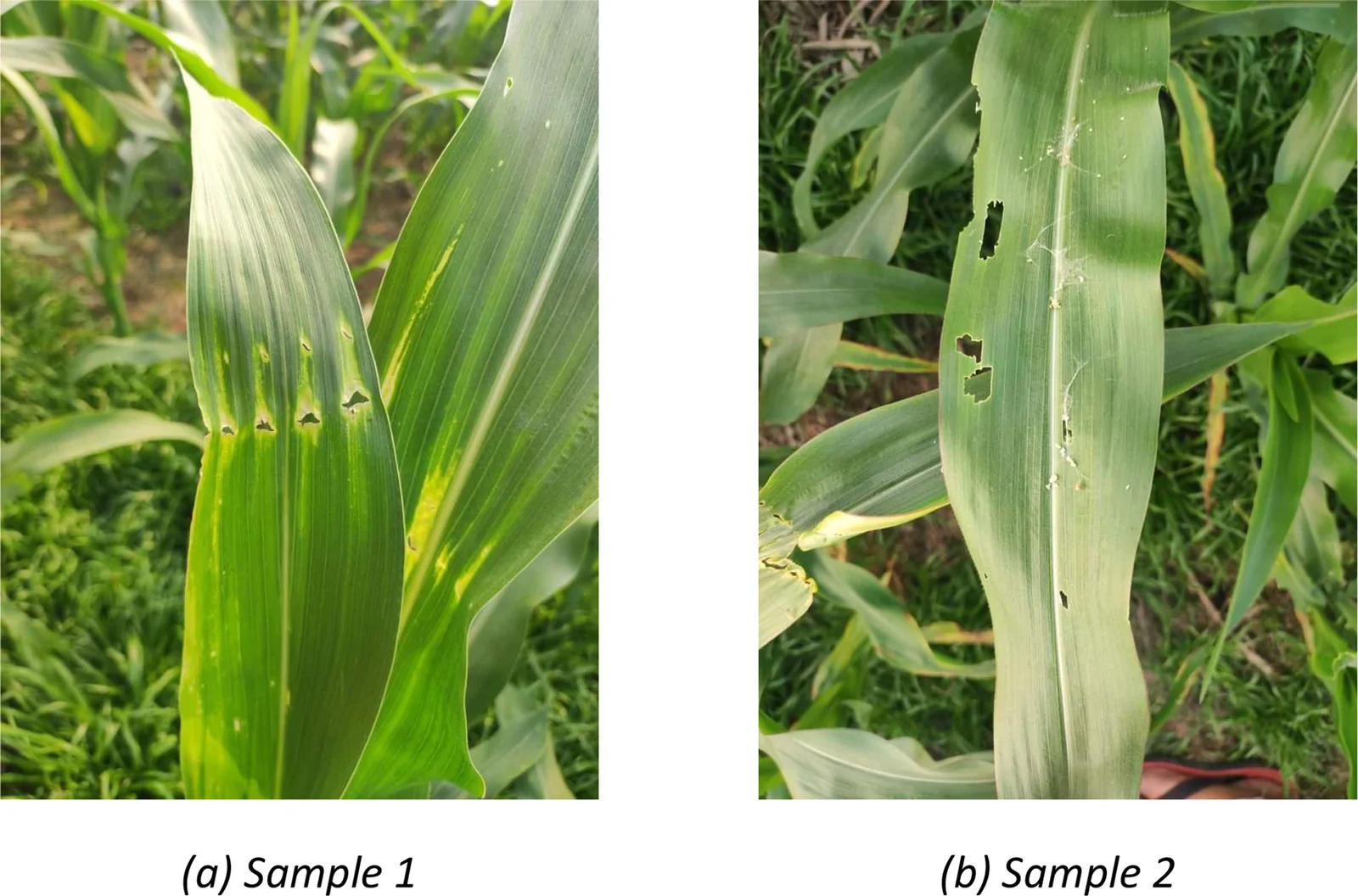
Fall Armyworm (Spodoptera frugiperda) feeding damage on maize leaves, showing characteristic ragged holes and window-pane scraping.

### 3.5 Leaf spot

Leaf Spot symptoms on maize can be produced by several fungal pathogens depending on prevailing weather and cultivar susceptibility, most commonly Curvularia lunata, Bipolaris maydis, and Cercospora species, all of which are established foliar pathogens of maize in South and Southeast Asia [7]. Spores of these fungi survive on crop residue between seasons and spread via wind and rain splash under warm, humid conditions, which favour rapid disease cycling. Infection produces discrete, roughly circular to oval necrotic spots on the leaf blade, typically pale tan to brown with a darker margin and sometimes surrounded by a fine chlorotic halo; under sustained humidity, individual spots may enlarge and coalesce into larger irregular patches, reducing the photosynthetically active leaf area. Unlike Northern Corn Leaf Blight, Leaf Spot lesions in this dataset are smaller, more rounded, and scattered across the blade rather than elongated and vein-parallel, which is the principal visual criterion used to distinguish the two classes during field labelling. Fig. 3 shows two representative Leaf Spot-affected leaves. This class comprises 407 images (10.2% of the dataset).

**Fig. 3:**
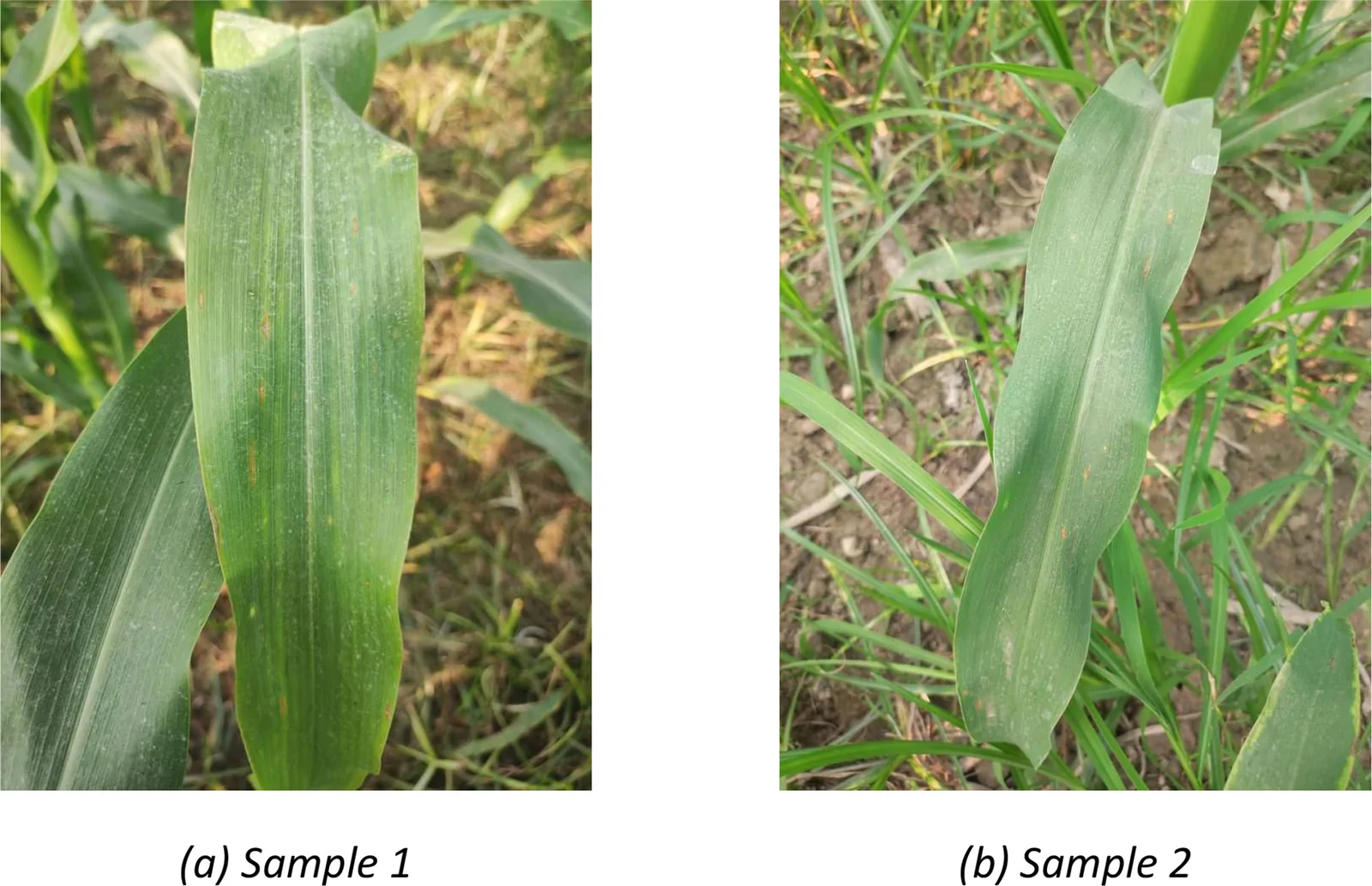
Leaf Spot symptoms on maize leaves, showing scattered necrotic lesions.

### 3.6 Northern corn leaf blight

Northern Corn Leaf Blight (teleomorph Setosphaeria turcica), one of the most economically important foliar pathogens of maize worldwide, including in South Asian production systems [6,7]. The pathogen overwinters on infected crop residue and is dispersed by wind-borne conidia, with disease severity favoured by moderate temperatures and prolonged leaf wetness. The disease produces long, elliptical, greyish-green to tan “cigar-shaped” lesions that run parallel to the leaf veins and can expand to several centimetres in length as the infection progresses; lesions typically appear first on the lower, older leaves and spread upward through the canopy as spores are dispersed by wind and rain splash. Because inaccurate or delayed diagnosis of NCLB and related foliar fungal diseases has been associated with yield losses exceeding 10% [7], and severe pre-flowering infection can reduce photosynthetic leaf area and grain yield substantially, prompt and correct identification is agronomically important. Fig. 4 shows two representative NCLB-affected leaves. This class comprises 535 images (13.5% of the dataset).

**Fig. 4:**
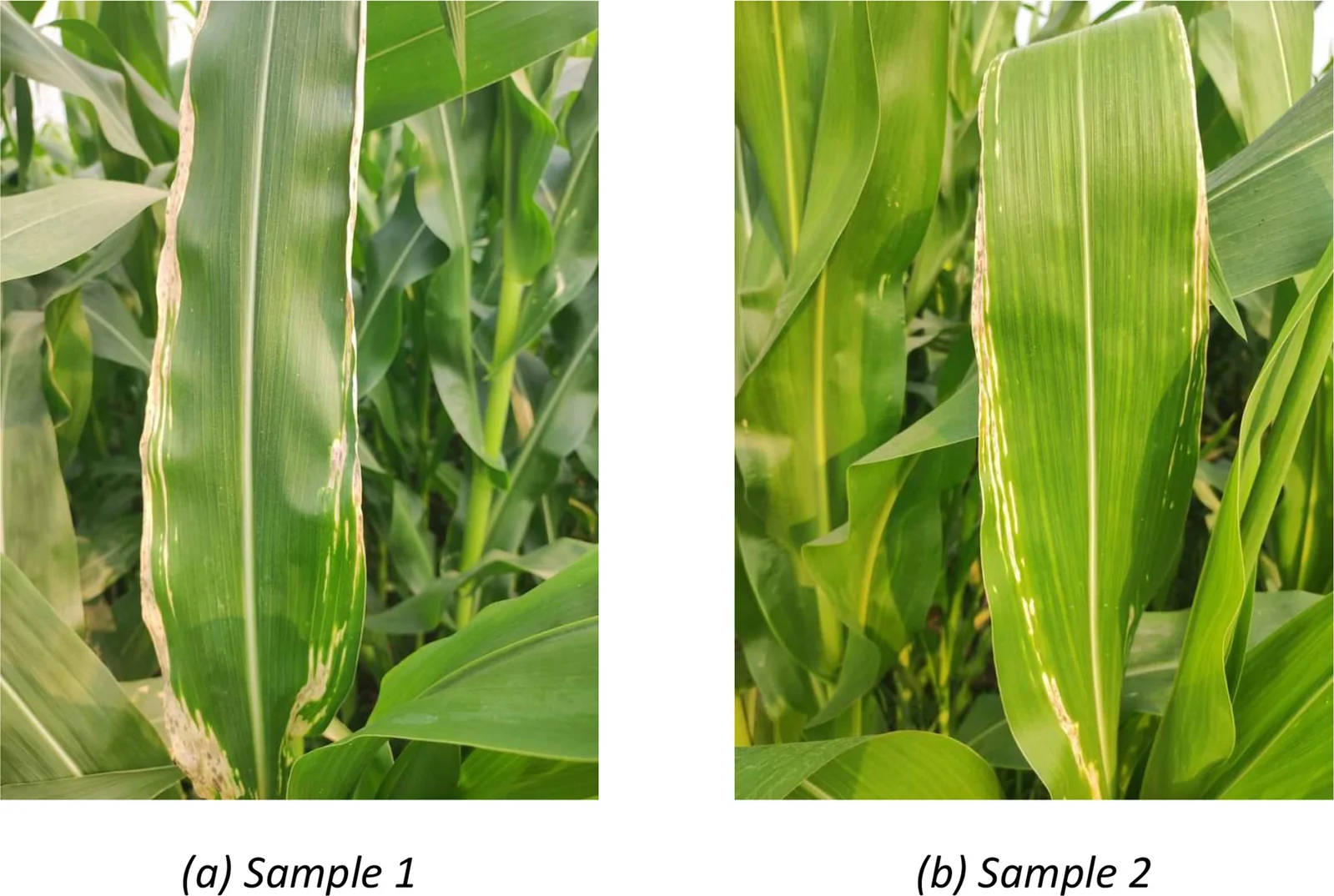
Northern Corn Leaf Blight (Exserohilum turcicum) lesions on maize leaves, showing elongated cigar-shaped necrotic streaks.

### 3.7 Nitrogen deficiency

Nitrogen is the nutrient most frequently limiting in maize production and is a cosre component of chlorophyll and amino acids required for vegetative growth; it is also the most easily leached macronutrient from sandy or intensively irrigated tropical soils. Because nitrogen is mobile within the plant, deficiency symptoms appear first on older, lower leaves as the plant remobilises available nitrogen toward actively growing tissue near the top of the canopy [8]. Affected leaves develop a characteristic V-shaped yellowing that begins at the leaf tip and spreads along the midrib toward the leaf base, while the leaf margins often remain green for longer than the interior of the blade, producing the distinctive V-shaped pattern used for field identification. Prolonged deficiency can cause premature senescence and death of the lower canopy, stunted growth, and reduced grain fill. Fig. 5 shows two representative Nitrogen-deficient leaves. This class comprises 425 images (10.7% of the dataset).

**Fig. 5:**
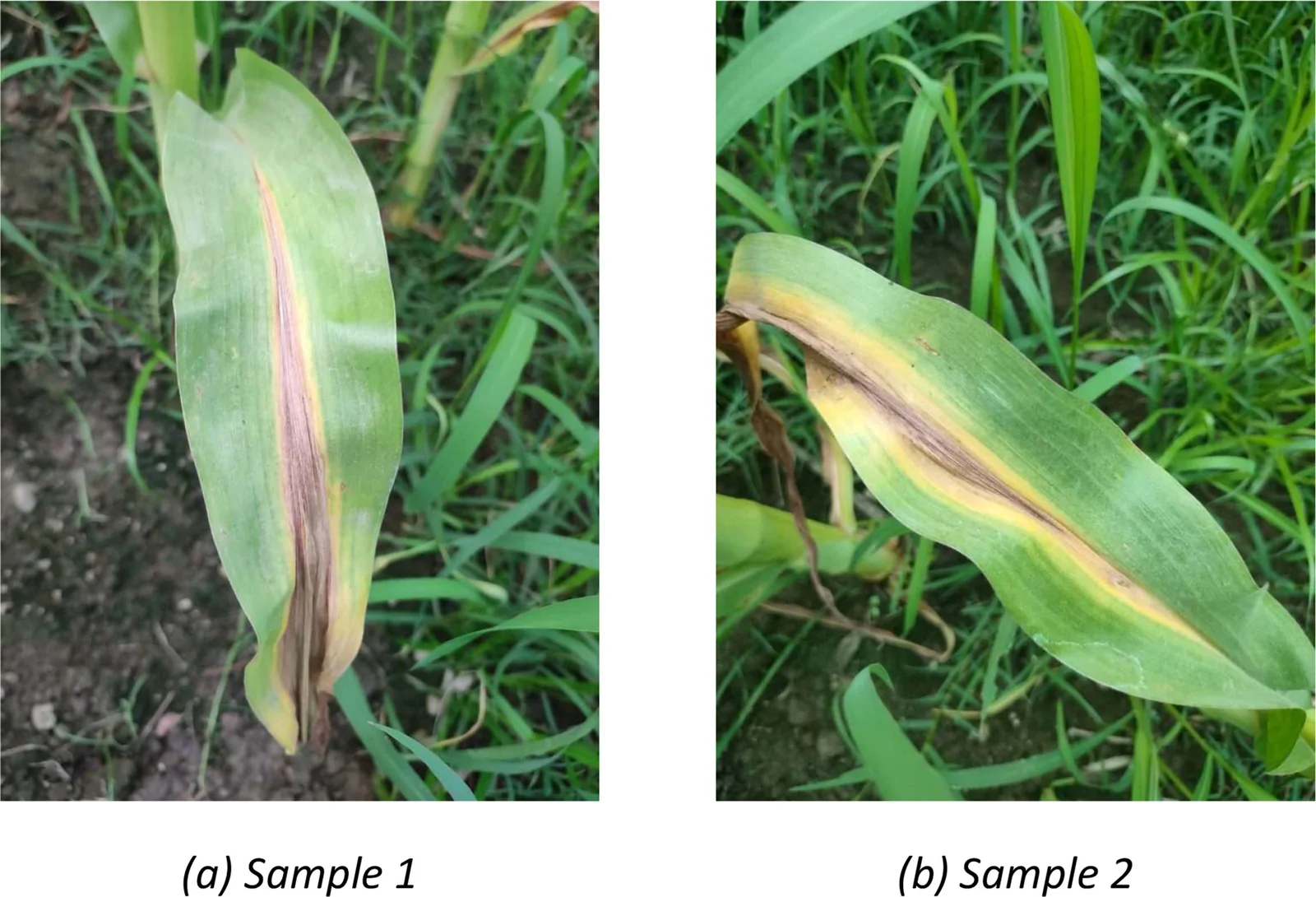
Nitrogen-deficient maize leaves, showing V-shaped yellowing along the midrib of older leaves.

### 3.8 Phosphorus deficiency

Phosphorus deficiency restricts energy transfer (ATP metabolism) and root development in maize and is particularly common on acidic, highly weathered tropical soils where phosphorus is readily fixed into plant-unavailable forms. Like nitrogen, phosphorus is mobile within the plant, so symptoms typically appear on older leaves first and progress upward if the deficiency is not corrected [8]. Affected leaves develop a reddish-purple to purplish-bronze discoloration, most pronounced along the leaf margins and tips, caused by the accumulation of anthocyanin pigments under phosphorus stress; affected plants may also appear stunted, with delayed maturity and reduced tillering. Because early-stage maize has a comparatively high phosphorus demand relative to its small root system, deficiency symptoms are especially likely to appear at the vegetative stage targeted by this dataset. Fig. 6 shows two representative Phosphorus-deficient leaves. This class comprises 500 images (12.6% of the dataset).

**Fig. 6:**
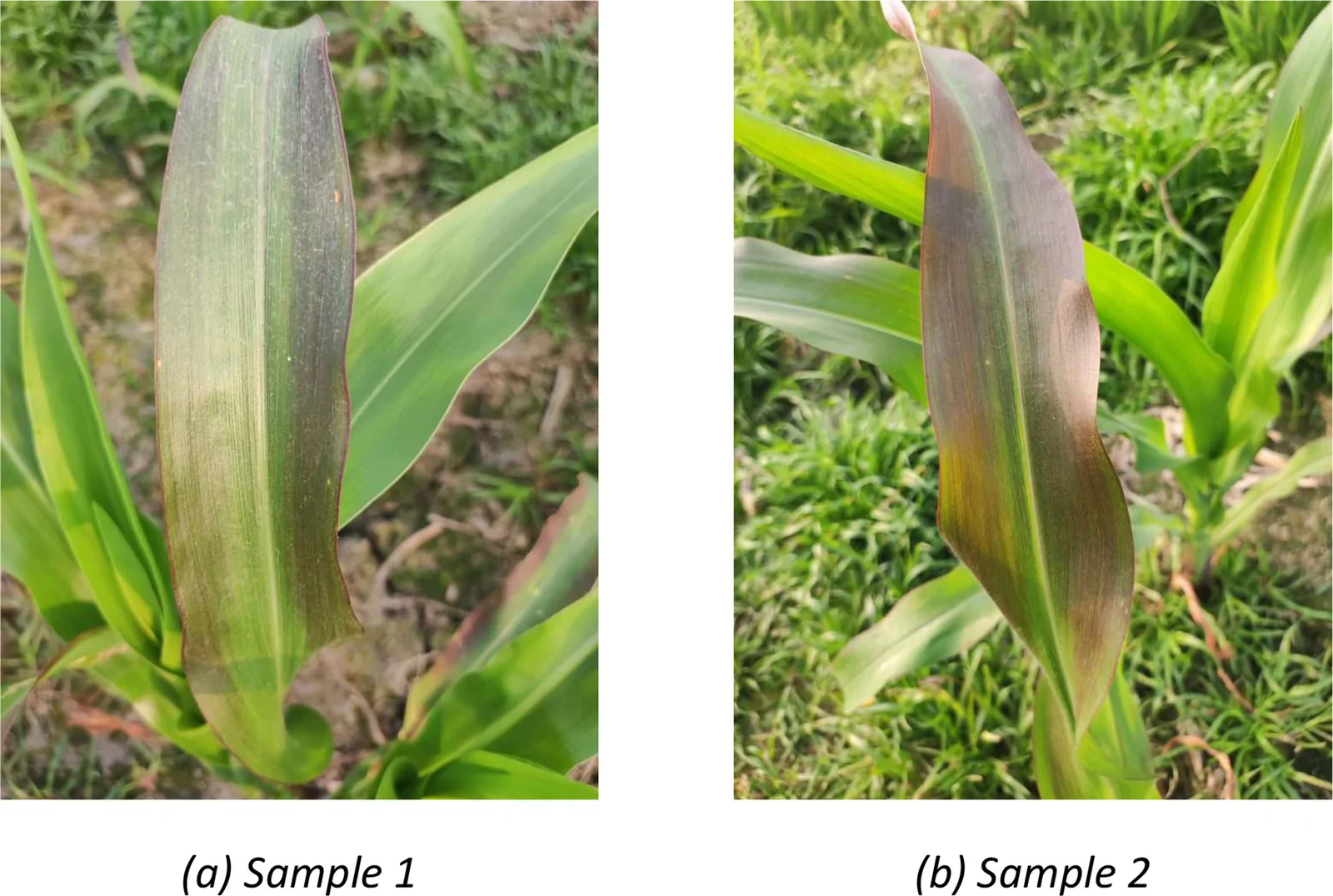
Phosphorus-deficient maize leaves, showing purplish-red discoloration most pronounced on older leaves.

### 3.9 Potassium deficiency

Potassium regulates stomatal function, water balance, enzyme activation, and stalk strength in maize and, like nitrogen and phosphorus, is mobile within the plant, so deficiency symptoms appear first on older leaves [8]. Characteristic symptoms include marginal chlorosis and necrosis (“scorching” or “firing”) that begins at the leaf tip and extends along the leaf edges, while the tissue nearer the midrib often remains green for longer; affected leaf margins may also curl or fray as the necrotic tissue dries. Because potassium supports stalk integrity and disease resistance more broadly, severely deficient plants are also more prone to lodging and secondary infection later in the season, compounding yield loss beyond the direct effect of the nutrient deficiency itself. Fig. 7 shows two representative Potassium-deficient leaves. This class comprises 210 images (5.3% of the dataset).

**Fig. 7:**
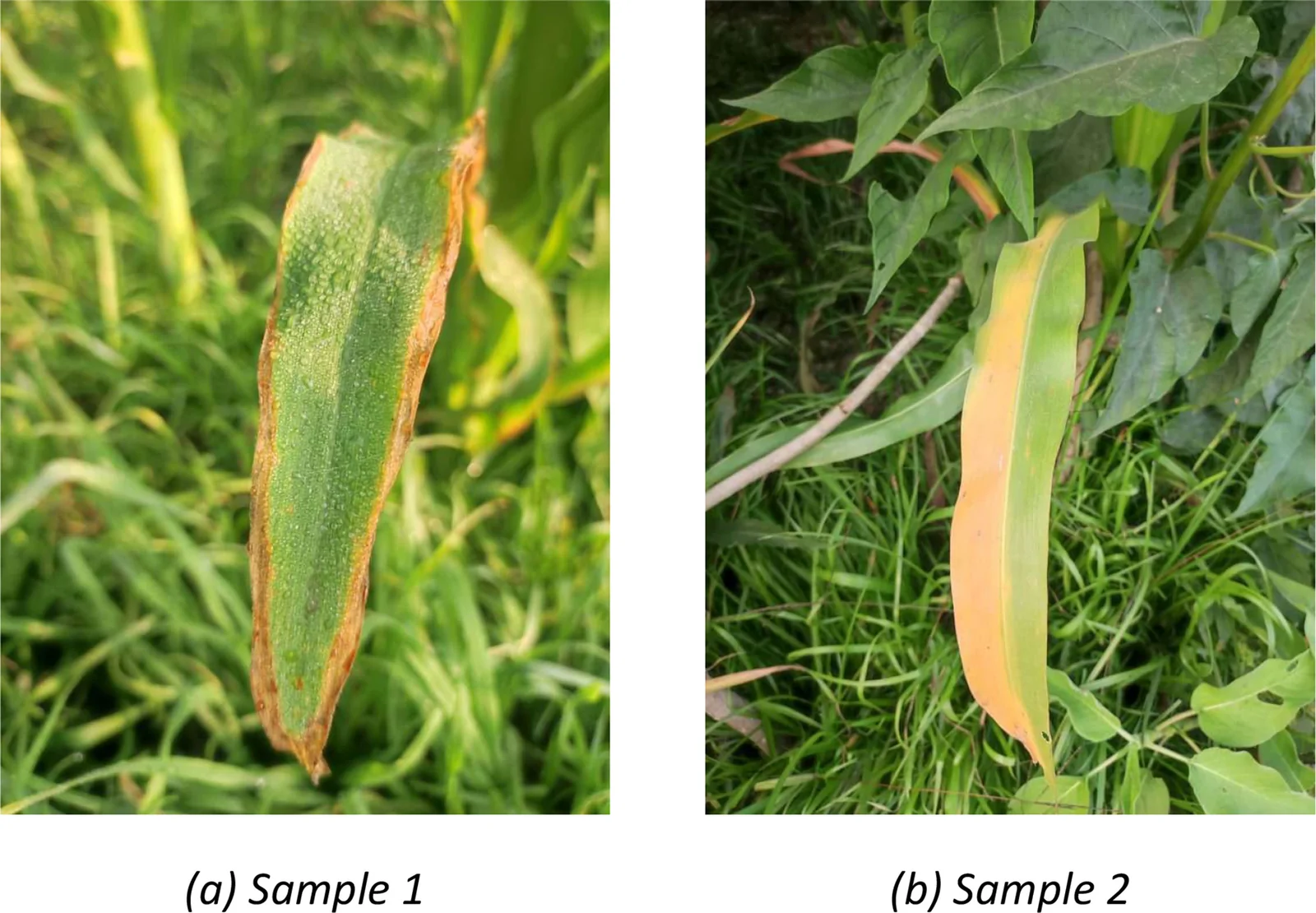
Potassium-deficient maize leaves, showing marginal scorching and necrosis on older leaves.

### 3.10 Magnesium deficiency

Magnesium is the central atom of the chlorophyll molecule, and its deficiency directly impairs photosynthetic capacity even before visible symptoms appear. As a mobile nutrient, deficiency symptoms first appear on older leaves as interveinal chlorosis, in which the tissue between the veins turns pale yellow to white while the veins themselves remain green, producing a distinctive striped or marbled appearance that is the primary field diagnostic for this class [8]. In advanced stages the interveinal tissue may develop reddish-purple pigmentation and necrosis, particularly on soils with inherently low magnesium availability or where an excess of potassium or ammonium antagonises magnesium uptake at the root. Fig. 8 shows two representative Magnesium-deficient leaves. This class comprises 553 images (13.9% of the dataset).

**Fig. 8:**
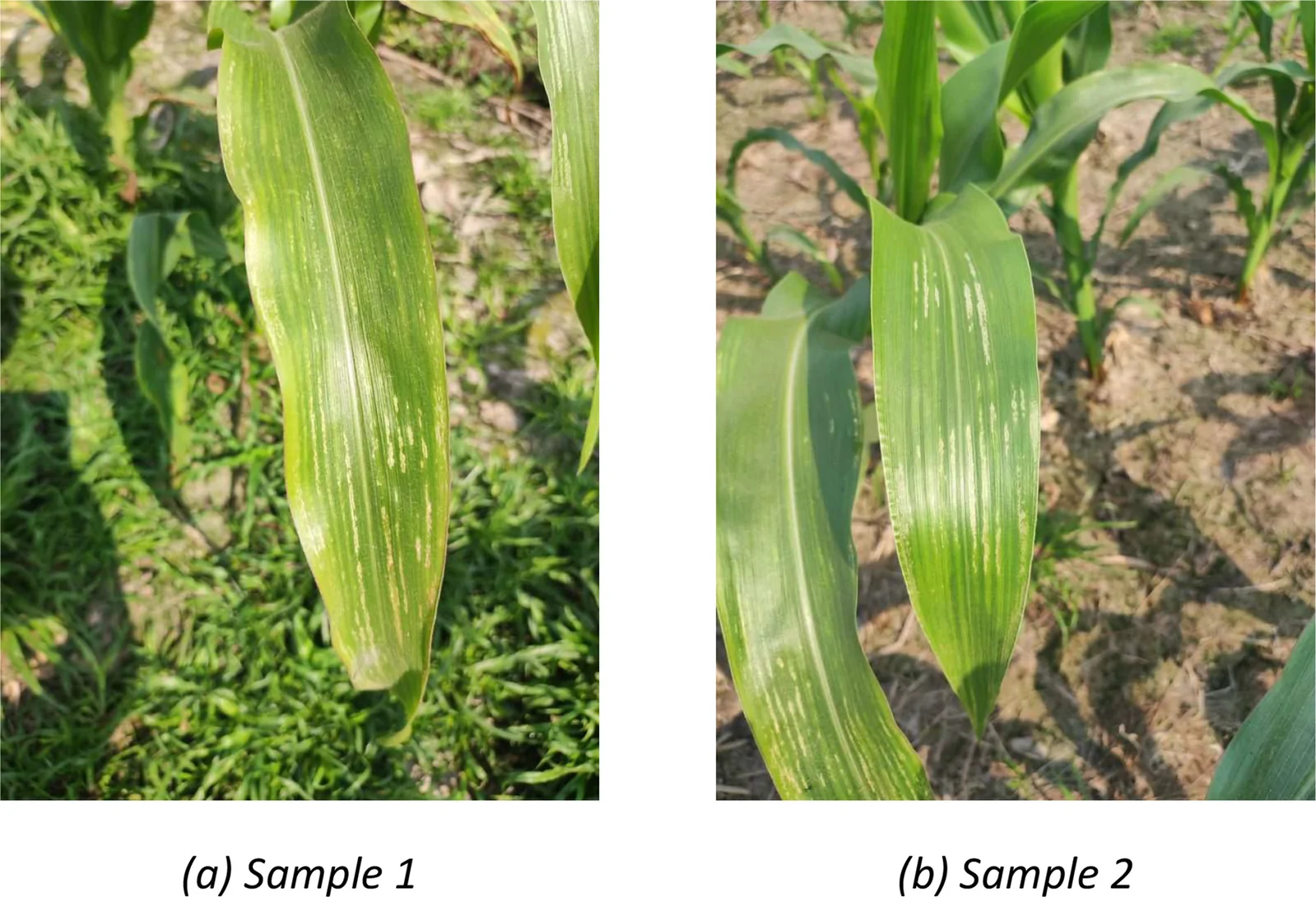
Magnesium-deficient maize leaves, showing interveinal chlorosis with veins remaining green.

### 3.11 File organisation and image properties

The released archive (FieldMaizeLeaf-8.zip, 26.4 GB) is organised into eight sub-folders, one per class, each named after its corresponding class (*Healthy_Leaf, Fall_Armyworm_Damage, Leaf_Spot, Northern_Corn_Leaf_Blight, Nitrogen_Deficiency, Phosphorus_Deficiency, Potassium_Deficiency, Magnesium_Deficiency*; full directory structure in Fig. 9 and Section 4.5). Within each folder, images are stored as individual JPEG files with sequential file names, with no further sub-division by field site or capture date. General attributes of the released image files including format, resolution, colour mode, and licence are summarised in Table 2, and all images were captured with the single smartphone device summarised in Table 3.

**Fig. 9:**
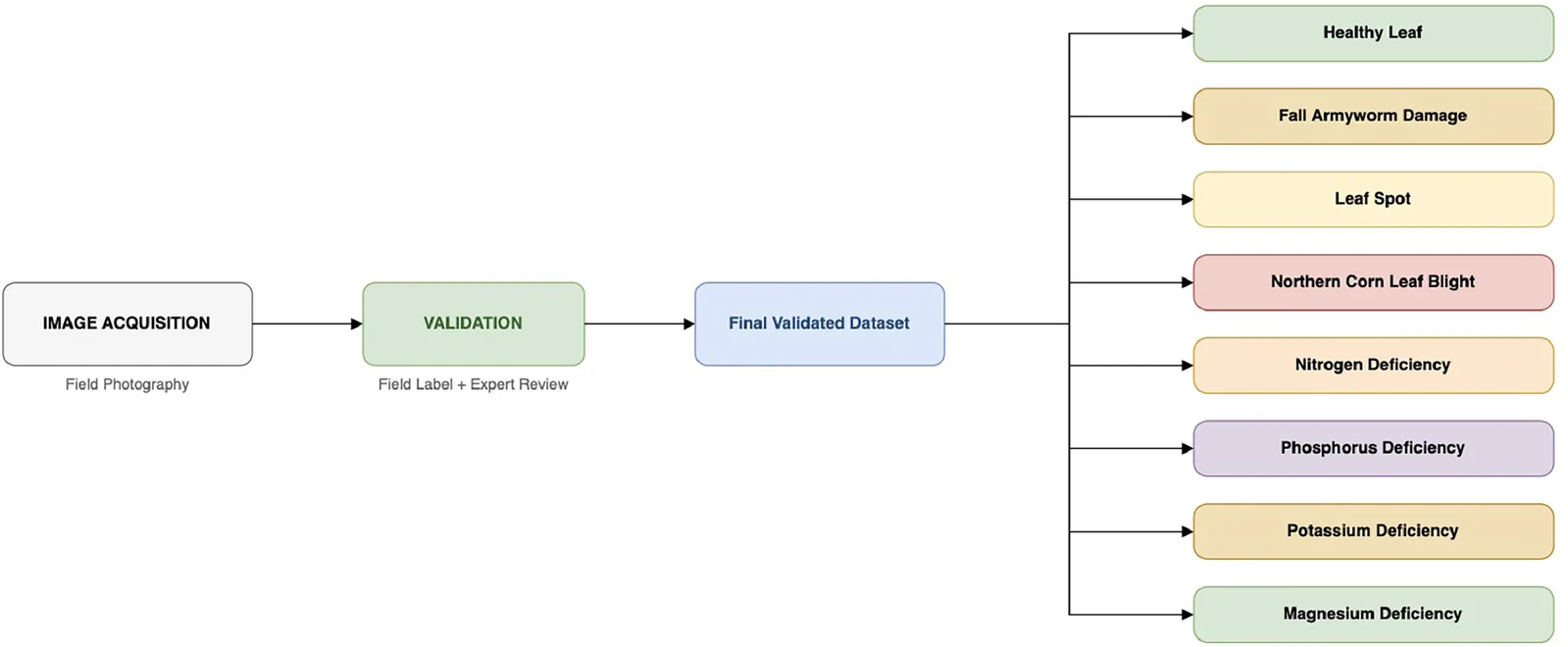
Workflow of the dataset collection, labelling, validation, and organisation process, showing the branching of the final repository into the eight released classes.

**Table 2:** General attributes of the released image files.

Attribute	Description
Total images	3975
Number of classes	8
Image format	JPEG
Color mode	RGB
Acquisition device	Xiaomi 11 Lite NE 5 G (rear camera, default camera application, no digital zoom)
Image resolution	Predominantly 3472 × 4640 pixels (16 MP binned output); a minority of images at other native resolutions (e.g., 1200 × 1600 pixels) depending on in-app camera settings
Archive size	26.4 GB (compressed)
License	Creative Commons Attribution 4.0 International (CC BY 4.0)

**Table 3:** Camera specifications of the acquisition device. The rear camera uses a 64-megapixel quad-Bayer sensor that outputs 16-megapixel binned JPEG images (3472 × 4640 pixels) in the default photo mode used for data collection; a small number of images were captured at other native resolutions (e.g., 1200 × 1600 pixels) depending on in-app settings at the time of capture.

ID	Product	Model	Megapixels	ImageSize	FileType
D1	Xiaomi	11 Lite NE 5G	64 (rear, quad-Bayer)	3472 × 4640 (16 MP binned output; typical)	JPEG

Table 4 situates FieldMaizeLeaf-8 relative to other publicly available plant-stress image datasets that are directly comparable in scope, either because they target maize specifically or because they follow a similarly structured, field-collected Data in Brief format. Unlike PlantVillage, which was collected under controlled laboratory conditions, or the Northern Leaf Blight image set, which targets a single maize disease, FieldMaizeLeaf-8 is, to the authors' knowledge, the first field-collected image dataset to combine multiple biotic and abiotic maize leaf-stress categories within one consistently labelled, expert-validated South Asian collection.

### 3.12 Comparison with related dataset

**Table 4:** Comparison of FieldMaizeLeaf-8 with related publicly available plant-stress image datasets.

Dataset	Image type	Classes	Images	Notes
PlantVillage [1]	Leaf, laboratory background	38 crop–disease classes (multiple crops, incl. maize)	∼54,000	Plain/uniform backgrounds; widely used but limited field realism
PlantDoc [2]	Leaf, field (web-sourced)	17 disease classes across 13 crop species	2598	Field images scraped from the internet; not maize-specific
Maize NLB image set [4]	Leaf, field/UAV	Northern Leaf Blight vs. healthy (1 disease)	18,222 annotated	Maize-specific but limited to a single disease; USA
FieldMaizeLeaf-8 (this study) [9]	Leaf, field (smartphone)	8 (Healthy + 3 biotic + 4 abiotic)	3975	Combines biotic and abiotic maize leaf stresses; Bangladesh; expert-validated

## 4 Experimental Design, Materials and Methods

The dataset was developed through five sequential stages: field photography, field-level class labelling, expert validation, data cleaning, and final organisation into the released repository. Fig. 9 illustrates this workflow, together with the branching of the validated dataset into its eight constituent classes.

### 4.1 Study area and crop stage

Data collection was carried out in commercial and smallholder maize fields in Daudkandi Upazila, Cumilla District, Chittagong Division, Bangladesh (approximately 23.53° N, 90.72° E), during the winter (rabi) maize-growing season, when maize is typically cultivated in the region between November and April [5]. Sampling targeted maize plants at the early vegetative growth stage, approximately 1–2 months after emergence, since this stage is agronomically important for the timely detection of both pest infestation and nutrient-deficiency disorders, before yield potential is compromised and while corrective management is still economically worthwhile.

### 4.2 Camera specification and acquisition protocol

All images were captured using the rear camera of a Xiaomi 11 Lite NE 5 G smartphone (Table 3) in its default camera application, with no macro lens attachment, external lighting, or laboratory staging. Photographs were taken handheld, outdoors, under ambient natural daylight, at distances and viewing angles representative of manual field scouting by a farmer or extension worker, rather than at a fixed standardised distance or angle. This acquisition protocol was chosen deliberately to preserve natural variability in illumination, background clutter, leaf orientation and symptom severity, so that the dataset better reflects the conditions under which a deployed field-diagnosis system would need to operate. Data collection took place between 25 January and 20 February 2026.

### 4.3 Class definition and field-level annotation

Each captured image was assigned in the field to one of eight classes based on the single dominant visible foliar symptom present on the photographed leaf: one healthy class (no visible symptoms) and seven stress classes covering three biotic conditions (Fall Armyworm damage, Leaf Spot, and Northern Corn Leaf Blight) and four abiotic nutrient-deficiency conditions (Nitrogen, Phosphorus, Potassium, and Magnesium deficiency). Field-level class assignment was based on standard, published visual symptom descriptions for maize foliar disease, pest damage, and nutrient deficiency [[5], [6], [7], [8]], summarised for each class in Section 3 together with the visual criteria (lesion shape, colour, and leaf position) used to distinguish visually similar classes from one another. Nutrient-deficiency classes were assigned on the basis of visible foliar symptoms only; no soil or leaf-tissue nutrient analysis was performed to confirm the underlying nutrient status (see Limitations).

### 4.4 Data quality control and expert validation

Following field collection, all images and their field-assigned class labels were reviewed by Sayed Mohammad Mohsin, Associate Professor, Department of Plant Pathology, Sher-e-Bangla Agricultural University, an expert in bsiotic and abiotic plant stress diagnosis. Each image was checked against the visual criteria summarised in Section 3, and the reviewer confirmed, corrected, or rejected the field-assigned label accordingly. Images with ambiguous or overlapping symptoms, severe motion blur or out-of-focus regions, near-duplicate framing of the same leaf, or symptoms inconsistent with the assigned class were relabelled where a confident alternative class could be determined, or otherwise excluded from the released dataset. This two-stage annotation workflow — non-expert field labelling followed by expert review — was intended to increase label reliability relative to field-only annotation while retaining the natural image-quality variability characteristic of real-field data collection, striking a balance between label accuracy and dataset realism.

### 4.5 Data organisation and release

The FieldMaizeLeaf-8 dataset is organized into eight class-specific directories corresponding to the maize leaf conditions represented in the collection. The folder names serve as the class labels, allowing the images to be directly loaded and processed using standard machine-learning and deep-learning frameworks.

The directory structure of the dataset is as follows:

FieldMaizeLeaf-8/

├—— Healthy Leaf/

├—— Fall Armyworm Damage/

├—— Leaf Spot/

├—— Northern Corn Leaf Blight/

├—— Nitrogen Deficiency/

├—— Phosphorus Deficiency/

├—— Potassium Deficiency/

└—— Magnesium Deficiency/

The complete archive (FieldMaizeLeaf-8.zip, 26.4 GB) was compressed and deposited in the Zenodo repository under a CC BY 4.0 licence, and assigned a persistent DOI to support long-term citability and reuse. No image augmentation, resizing, or other pixel-level pre-processing was applied prior to release; images are provided exactly as captured by the acquisition device, so that end users can apply whatever resizing, normalisation, or augmentation pipeline best suits their own modelling approach.

## Limitations

Data were collected at a single site (Daudkandi, Cumilla) during a single growing season and a single ∼4-week collection window, so the dataset does not capture inter-annual, inter-regional, or multi-device variability, and models trained solely on it may not generalise fully to other cultivars, soils, or climates. Nutrient-deficiency class labels are based on visual foliar symptoms only and were not confirmed by soil or leaf-tissue nutrient analysis; visually similar symptoms can, in principle, result from more than one underlying nutrient deficiency, from co-occurring water stress, or from early disease symptoms that mimic nutrient chlorosis [8]. Class sizes are imbalanced, ranging from 210 images (Potassium Deficiency) to 849 images (Fall Armyworm Damage), which may require class-balancing strategies during model training. All images were captured with a single smartphone model, and labels are image-level only, without bounding-box, segmentation, or symptom-severity-grade annotations, which limits the dataset's direct use for detection or severity-estimation tasks without further annotation.

## Ethics Statement

The authors have read and followed the ethical requirements for publication in Data in Brief. This work does not involve human subjects, personally identifiable information, animal experiments, or data collected from social media platforms; the dataset consists exclusively of photographs of maize (Zea mays L.) leaves. Field access for data collection was obtained with the permission of the respective field owners.

## Credit Author Statement

• **Laboni Akter**: Conceptualization, Methodology, Data Collection, Investigation, Data curation, Writing – original draft.
• **Md Shohel Arman:** Conceptualization, Methodology, Supervision, Project administration, Writing – review & editing.
• **Sakibul Hasan Chowdhury:** Data curation, Formal analysis, Visualization, Conceptualization, Methodology, Writing – review & editing.
• **Sayed Mohammad Mohsin:** Validation, Methodology

## Data Availability

ZenodoFieldMaizeLeaf-8: A Real-Field Early Vegetative Maize Leaf Stress Image Dataset from Bangladesh (Original data)
